# Genotypic Prediction of Tropism of Highly Diverse HIV-1 Strains from Cameroon

**DOI:** 10.1371/journal.pone.0112434

**Published:** 2014-11-07

**Authors:** Christelle Mbondji-Wonje, Viswanath Ragupathy, Jiangqin Zhao, Aubin Nanfack, Sherwin Lee, Judith Torimiro, Phillipe Nyambi, Indira K. Hewlett

**Affiliations:** 1 Laboratory of Molecular Virology, Division of Emerging and Transmission Transmitted Diseases, Office of Blood Review and Research, Center for Biologics Evaluation and Research, Food and Drug Administration, 10903 New Hampshire Avenue, Silver Spring, MD, 20993, United States of America; 2 Faculty of Medicine, Pharmacy and Biomedical sciences, University of Douala, Douala, Cameroon; 3 Chantal Biya International Reference Centre, Yaoundé, Cameroon; 4 Department of Pathology, New York University School of Medicine, New York, New York, United States of America; Institute of Infection and Global Health, United Kingdom

## Abstract

**Background:**

The use of CCR5 antagonists involves determination of HIV-1 tropism prior to initiation of treatment. HIV-1 tropism can be assessed either by phenotypic or genotypic methods. Genotypic methods are extensively used for tropism prediction. However, their validation in predicting tropism of viral isolates belonging to group M non-B subtypes remains challenging. In Cameroon, the genetic diversity of HIV-1 strains is the broadest reported worldwide. To facilitate the integration of CCR5 antagonists into clinical practice in this region, there is a need to evaluate the performance of genotypic methods for predicting tropism of highly diverse group M HIV-1 strains.

**Methods:**

Tropism of diverse HIV-1 strains isolated from PBMCs from Cameroon was determined using the GHOST cell assay. Prediction, based on V3 sequences from matched plasma samples, was determined using bioinformatics algorithms and rules based on position 11/25 and net charge applied independently or combined according to Delobel's and Garrido's rules. Performance of genotypic methods was evaluated by comparing prediction generated with tropism assigned by the phenotypic assay.

**Results:**

Specificity for predicting R5-tropic virus was high, ranging from 83.7% to 97.7% depending on the genotypic methods used. Sensitivity for X4-tropic viruses was fairly low, ranging from 33.3% to 50%. In our study, overall, genotypic methods were less able to accurately predict X4-tropic virus belonging to subtype CRF02_AG. In addition, it was found that of the methods we used the Garrido rule has the highest sensitivity rate of over 50% with a specificity of 93%.

**Conclusion:**

Our study demonstrated that overall, genotypic methods were less sensitive for accurate prediction of HIV-1 tropism in settings where diverse HIV-1 strains co-circulate. Our data suggest that further optimization of genotypic methods is needed and that larger studies to determine their utility for tropism prediction of diverse HIV-1 strains may be warranted.

## Introduction

Human immunodeficiency virus type 1 (HIV-1) enters host cells in a multistep process that involves the binding of the viral gp120 protein to the CD4 receptor and chemokine receptors CCR5 and CXCR4 [1], [2]. HIV-1 isolates gaining entry into the cell through the CCR5 co-receptor are classified as R5 tropic viruses. Similarly isolates that use the CXCR4 co-receptor are classified as X4 tropic viruses. Certain viruses use both co-receptors to enter the host cell and are referred as dual/mixed tropic viruses or R5X4 viruses. The use of both co-receptors may be due to the presence of either dual clones or a mixture of pure R5 and X4 virus clones or both in the viral population in a given sample [3].

At the present time CCR5 antagonists are the only widely available entry inhibitors. In clinical practice, they have been proven to be effective in patients harboring exclusively R5-tropic viruses and may not be effective against X4-tropic or dual/mixed HIV-1 strains [4]–[6]. Therefore, determining tropism of the patient's HIV strain is necessary prior to using CCR5 antagonists in HIV treatment regimens. HIV-1 tropism can be evaluated either by cell-based phenotypic assays or predicted by nucleotide sequence-based genotypic methods. Although phenotypic assays are the most reliable means to assess HIV-1 tropism, they are expensive, laborious and require special facilities and expertise. Thus, genotypic methods have been proposed as an alternative as they are less time consuming and expensive. Genotypic methods are based on analysis of the nucleotide sequence of the V3 region of the gp120 envelope protein. Genotypic prediction of tropism relies on bioinformatics algorithms and/or characteristics of the V3 sequence such as positive charges at positions 11 and/or 25, global net charge and the loss of potential N-linked glycosylation sites [7]–[9]. Paired genotypic/phenotypic data generated from comparative studies have led to the validation of genotypic approaches in predicting tropism of HIV-1 subtype B [10]. However, the utility for non-B subtypes remains uncertain due to the lack of sufficient datasets. In addition, accurate prediction of tropism of non-B strains is complicated by the high genetic variability within the V3 region [11], [12].

Cameroon, a country in West Central Africa where multiple subtypes, circulating recombinant forms (CRFs) and unique recombinant forms (URFs) have been reported, hosts broad HIV-1 genetic diversity [13]–[18]. Regional co-circulation of divergent HIV-1 variants has an impact on the global HIV-1pandemic. Non B subtypes and CRFs were originally identified in West Central Africa and increased global travel and immigration have contributed to their spread. These diverse non B subtypes and CRFs are responsible for about 90% of current HIV-1 infections worldwide [19], [20]. Indeed, increasing cases of HIV-1 infection with non-B subtypes have been reported in Europe and North-America [21]–[24]. According to Brennan et al, group M infections account for 97.3% of HIV-1 cases in Cameroon and more than 99% of them are due to non-B subtypes [13]. In Cameroon, as in many other countries in Sub-Saharan Africa, introduction of CCR5 antagonists into clinical practice is anticipated in the future. To facilitate their appropriate incorporation into antiretroviral treatment, there is an urgent need to investigate the tropism of diverse HIV-1 strains and the use of genotypic methods to predict their tropism accurately. Countries in West Central Africa such as Cameroon may benefit from using genotypic methods as they are less labor-intensive and potentially more cost-effective. Considering that co-circulation of multiple HIV-1 subtypes may present a major challenge for tropism determination in this region, it is important to address the suitability of genotypic tools to predict tropism of HIV-1 strains circulating in this region of Africa.

The primary aim of this study was to assess the ability of genotypic methods to predict co-receptor usage of HIV-1 variants circulating in Cameroon.

## Material and Methods

### Ethics statement

The study was approved a FDA Institutional Review Board, the US HHS/Food and Drug Administration Research in Human Subjects Committee and the exempt reference number is 01-044B. Specimens used had all been previously obtained for another purpose and were de-identified for the purpose of this study.

### Samples specimens

Paired PBMC and plasma specimens from HIV-1 infected patients from urban areas in Cameroon were analyzed. Paired specimens were collected at the same time point for each patient. Overall, the median age of the study population was 27 (IQR: 10, 35) and 59% were female.

### Culture of Primary HIV-1 isolates

Primary HIV-1 isolates were obtained by co-cultivation of patient PBMCs with PHA-stimulated PBMCs from HIV-1 negative healthy donors as previously described [25].The growth of virus was monitored using a p24 antigen enzyme-linked immunosorbent assay (ELISA) from Perkin Elmer (Cat No: NEK050B) performed according to the manufacturer's instructions. Culture supernatants of co-cultures that were identified as positive for HIV by p24 antigen ELISA were retested to confirm the amounts of antigen, and virus cultures were harvested and stored at −70°C until further use.

### Phenotypic characterization of co-receptor usage

Phenotypic characterization of co-receptor usage was determined by infecting GHOST cells that expressed either CD4 alone (parental) or CD4 along with either CCR5 or CXCR4. Briefly, GHOST-CCR5, GHOST-CXCR4 and parental GHOST cells were seeded in 24-well plates with 2.5×10^4^ cells per well in 1 mL of Dulbecco's modified Eagle's medium (DMEM) supplemented with 10% fetal calf serum (FBS). After 24 hours, the medium was removed and replaced with 0.5 mL of fresh medium containing the primary isolate (5 ng of p24 antigen). After 2 hours of incubation at 37°C with 5% CO2, virus containing media was removed and fresh DMEM medium supplemented with 10% FBS with antibiotics. Positive and negative control viruses for each co-receptor were included in the assay. After 4 to 7 days of infection, GFP expression was observed by fluorescence microscopy. Culture supernatants were harvested at days 7 and HIV replication was determined by measuring p24 antigen levels using the Perkin Elmer kit.

### RNA isolation, cDNA synthesis and PCR amplification

Isolation of viral RNA from plasma specimens was performed by using Virus QIAamp Viral RNA Mini Kit (QIAGEN) according to manufacturers' instructions. cDNA was obtained by using Superscript III First-strand synthesis System for RT-PCR (Invitrogen 18080-051) in a 20 µl volume with in-house primers that have been previously published [26]. Amplification was performed by a nested polymerase chain reaction (nested-PCR) with ready-to-use Master Mix from Promega (Promega, Madison, WI, USA cat M7505). Each run was performed in a 30 µl PCR mixture which included 0.4 µM of each outer (forward HXB2 coordinate 6203–6224 and reverse primer HXB2 coordinate 7514–7531) and an inner primer (forward HXB2 coordinate 6322–6350) previously published [27]. PCR cycling conditions were as follows: initial denaturation at 94°C for 2 min followed by 30 cycles (94°C, 30 s; 55°C, 30 s; 68°C, 80 s) and a final extension step at 68°C for 10 min. Each 1.2 kb amplicon was purified using the ExoSAP-IT (USB, Cleveland, Ohio, USA, cat 78200). After purification, samples were sequenced by the DNA sequencing core facility of CBER/FDA.

### Phylogenetic analysis

All retrieved sequences were loaded and assembled into vector NTI advance 11 software (Invitrogen) to generate contigs. Sequences were aligned with identical regions of reference sequences of known HIV-1 group M (sub-) Subtypes/CRFs 2013 reference sequences from the Los Alamos database (http://www.hiv.lanl.gov/content/sequence/NEWALIGN/align.html) using CLUSTALW [28] Phylogenetic analyses were conducted using MEGA version 5 [29] and trees were constructed from the generated sequences by the neighbor-joining method. Clustering of sequences with a bootstrap value of more than 70% was used to determine the HIV-1 subtype.

### Genotypic prediction of HIV-1 tropism

Genotypic prediction of HIV-1 tropism was based on the env-V3 loop amino acid sequences retrieved from the translation of nucleotide sequences into their protein equivalent using Transeq [30]. CCR5 (R5-tropic) and CXCR4 (X4-tropic) genotype were identified by using bioinformatics algorithms Geno2pheno (G2P) (http://co-receptor.bioinf.mpisb.mpg.de/cgi-bin/co-receptor) and WebPSSM (http://ubik.microbiol.washington.edu/computing/pssm/). Predictions by Geno2pheno were done with false positive rates (FPR) chosen at 5% (G2P-FPR5), 10% (G2P-FPR10) and 15% (G2P-FPR15). Predictions with WebPSSM were done by using the two subtype B matrices (PSSMsinsi and PSSM_X4R5_). Tropism prediction was performed either by applying rules separately or in combination. This included a) the 11/25 rule (11/25) for which positively charged amino acids (R or K) at either position 11 and 25 of the V3 region are required to predict an X4-tropic virus and b) the net charge rule for which a global net charge of the V3 ≥5 to predict an X4-tropic virus. The net charge of the V3 sequence was determined using the peptide-property calculator from Innovagen (http://www.innovagen.se).

The other approaches were a) - the Delobel rule for which one of the following criteria should be met for predicting X4-tropic virus: 11 R/K and/or 25K; 25R and a net charge ≥ +5; or a net charge ≥ +6 [31] and b) the Garrido rule which predicts X4-tropic if any of the 11/25 or Net charge rules classified it as X4 [32].

### Statistical analysis

In this study, the phenotypic GHOST cell assay was taken as the reference test to determine viral tropism. Specificity rate for R5 prediction was obtained from the ratio of samples predicted as CCR5 of the total CCR5 tropic samples in GHOST cell assay. Sensitivity rate for X4 prediction was obtained from the ratio of samples predicted as X4 over the total CXCR4 using samples in the GHOST cell assay.

## Results

### Phenotypic characterization of co-receptor usage

Of the 95 PBMC samples selected for this study, GHOST cell infectivity could be successfully performed for 62 isolated viruses (65.26%). The failure of virus isolation and expansion of the remaining samples was likely due to the unavailability of sufficient viable HIV-1 infected cells in the sample. Our experiments revealed that 49 (79.03%) virus isolates were able to infect GHOST cells expressing CCR5 co-receptor, while 8 (13%) infected GHOST cells expressing CXCR4 co-receptor exclusively. Five of the 62 (8%) viruses were able to infect cells that expressed both CCR5 and CXCR4 co-receptors.

### Phylogenetic analysis

**Figure 1:**
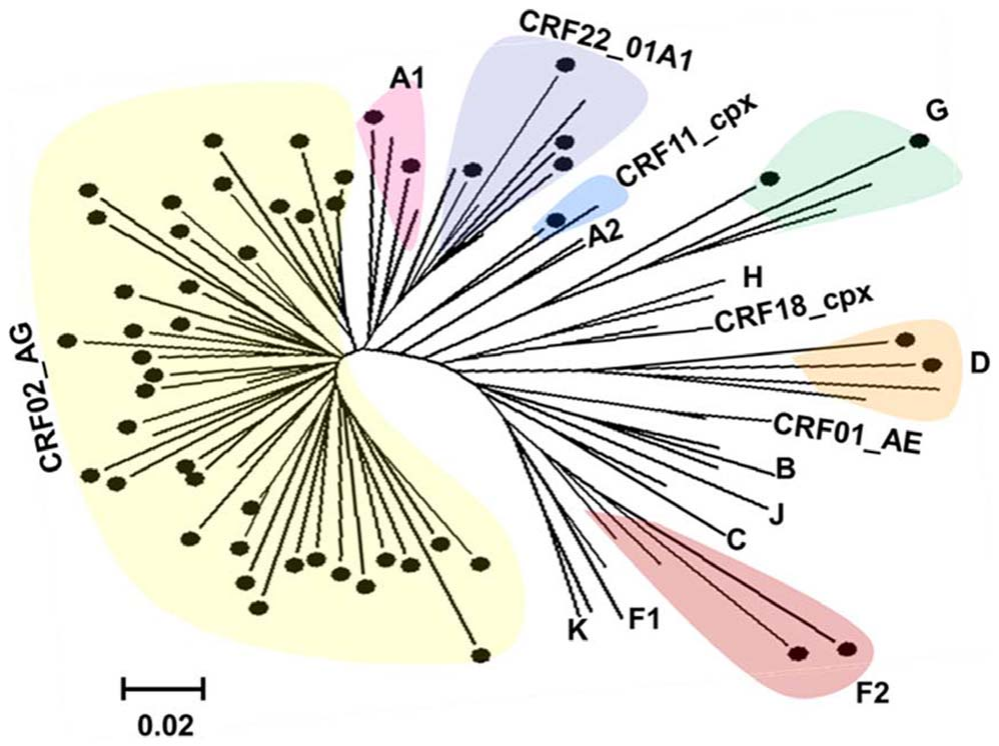
Phylogenetic analysis of the HIV-1 partial env region genome sequence. Analysis was performed using the neighbor-joining methods in Mega 5 program. Thereference subtypes and CRFs were used to construct the tree, and some references have been omitted for clarity. Bootstrap value above was 70%. The scale bar represents 2% genetic distance. The identified HIV-1 strains are indicated as full black circle “•”.

In the matched plasma samples, partial env sequences were successfully obtained for 55 (88.7%) out of the 62 phenotyped specimens. Overall, within the 55 sequences generated, 7 HIV-1 subtypes were identified. The genetic classification of these subtypes is shown in Figure 1. Briefly, 41 (74.5%) of the samples analyzed were from patients infected with subtype CRF02_AG and 14 (25.5%) from patients infected with non-CRF02_AG strains (distributed as follows: 5 CRF22_01A1 (9.1%), 2 F2 (3.6%), 2 A1 (3.6%), 2 G (3.9%), 2 D (3.6%) and 1 CRF11_cpx (1.8%)).

### Genotypic prediction of co-receptor usage

**Figure 2:**
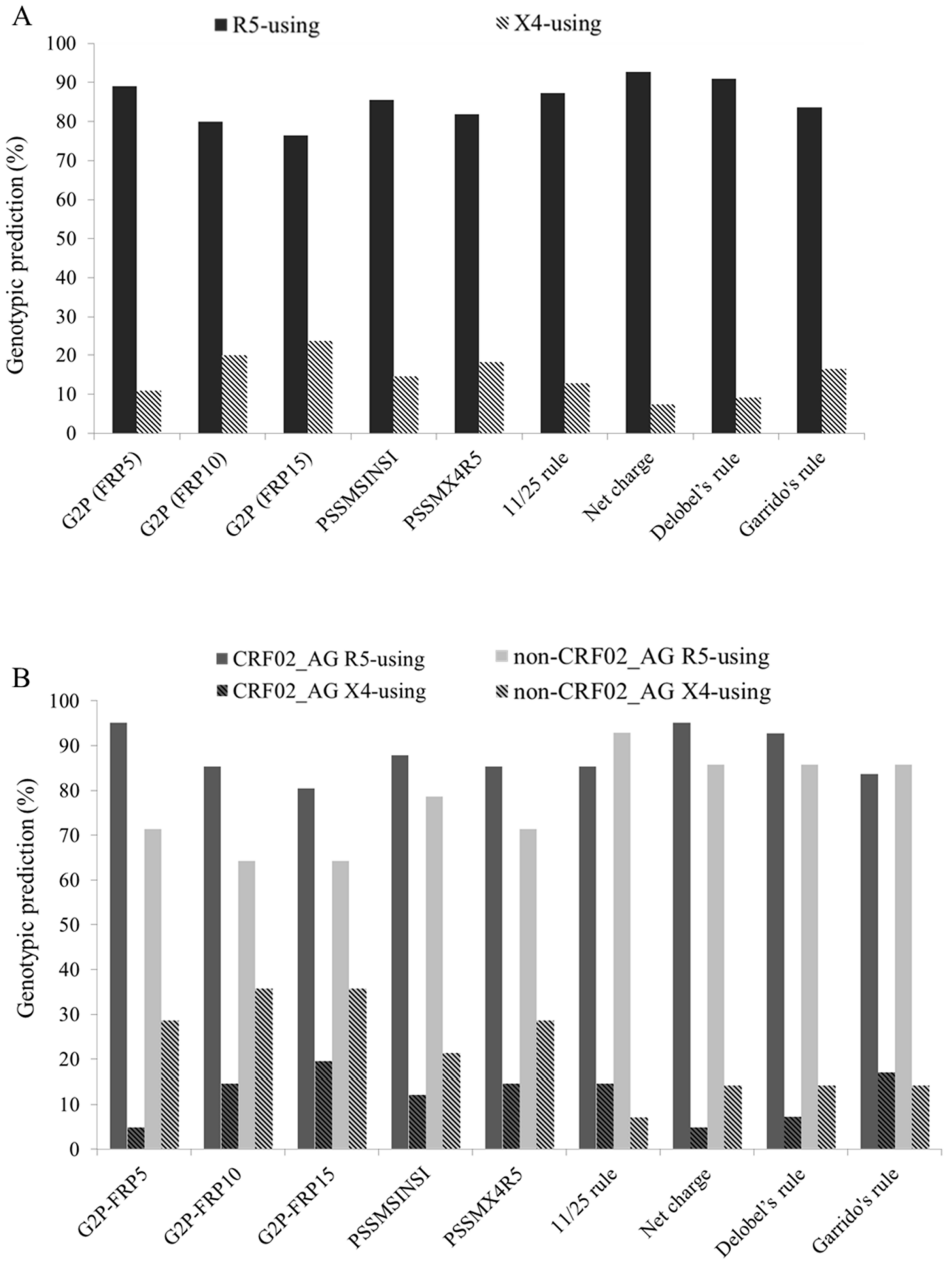
Prediction of HIV-1 co-receptor usage by using genotypic methods. (A) Prediction was assessed for the total number of samples. (B) Prediction was assessed in samples infected with HIV-1 subtype CRF02_AG or non-CRF02_AG strains.

While phenotypic results could be obtained from 62 patient PBMCs, only 55 matched plasma specimens gave a readable V3 sequence. Failure of amplification and/or sequencing of the remaining samples (11.3%) were probably due to the high genetic variability within the envelope region. As genotypic approaches do not discriminate viruses using exclusively CXCR4 co-receptor (X4-exclusive) from R5X4 viruses, they were both predicted as X4-tropic viruses. Genotypic prediction of co-receptor usage is shown in figure 2A. Briefly, using G2P, according to the specific FPR criteria, the range of R5-tropic viruses was between 76.4% and 89.1%; while X4-tropic viruses were predicted in 6 (10.9%) to 13 (23.6%) samples of the 55 analyzed. With PSSM, depending on the matrix used, R5 tropism was assigned in 45 (81.8%) to 47 (85.5%) of the 55 samples. By using predictive rules based on the presence of a basic amino-acid residue at position 11 and/or 25 or the net charge of the V3 region, R5- tropic virus was predicted in 48/55 (87.3%) and 51/55 (92.7%) samples, respectively. According to the net charge, only 4/55 (7.3%) of the samples had an X4-tropic virus. The proportion of samples predicted to harbor an R5-tropic virus was 83.6% with the Garrido rule and 90.9% with the Delobel rule (figure 2A).

When prediction of co-receptor usage was assessed according to HIV-1 subtype, for all the predictive tools, with the exception of G2P-FPR5 (p<0.05), we found that proportions of viruses predicted to be R5 tropic among CRF02_AG strains and among non-CRF02_AG strains were comparable. However, the proportion of viruses predicted to be X4-tropic was almost two times higher within non CRF02_AG subtype compared with CRF02_AG. The 11/25 rule was the only method which predicted higher rates of X4 tropism among samples harboring CRF02_AG (figure 2B).

### Correlation between phenotypic and genotypic predictions of co-receptor usage

**Figure 3:**
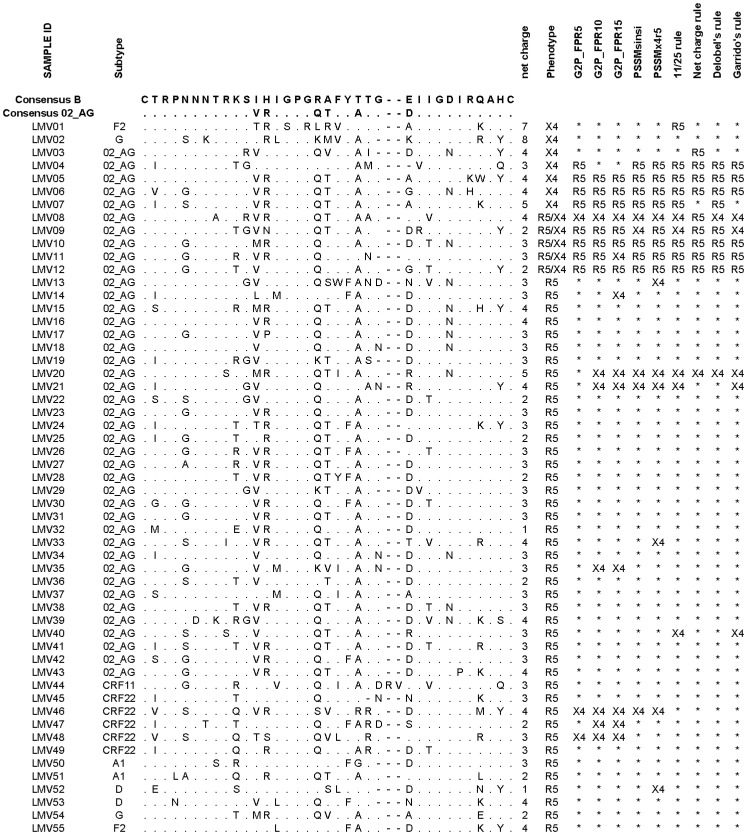
Alignment of V3 sequences from HIV-1 infected plasma from Cameroon. Predictions by different algorithms were compared with phenotype characterization. Concordant genotypic predictions of R5-tropic (R5) or X4-tropic (X4) viruses with virus phenotype were indicated with an asterisk (*).

Next we measured phenotypic predictions for matched PBMC viruses of a set of patient plasma specimens. Paired phenotypic and genotypic results were available for 55 samples. Of these, 43 (78.2%) were shown to harbor viruses with an exclusive CCR5 phenotype, five (9.1%) harbored a dual/mixed virus population and seven samples (12.7%) were infected with a virus using exclusively CXCR4 co-receptor (X4-exclusive). Of the 55 samples, 41 were from patients infected with HIV-1 CRF02_AG. Among them, 31 (76%) of the virus population showed an R5 phenotype, while the X4 and R5X4 phenotypes were present at the same rate of 12% (figure 3). Dual/mixed tropic viruses were mostly (4/5) reported as R5-tropic viruses with predictive tools.

Sensitivity and specificity of each genotypic algorithm was calculated according to previously described criteria and are presented in table 1. Specificity rates of genotypic methods varied between 83.7% and 97.7%. Sensitivity to predict X4-tropic viruses was fairly low, ranging from 33.3% to 50%. Sensitivity of each method generally increased when R5/X4 viruses were excluded from the analysis, except for the 11/25 rule (table 1). G2P-FPR5, net charge and Delobel's rule displayed a specificity over 95% with a sensitivity up to 42.9%, when dual/mixed were excluded. G2P-FPR15 and Garrido's rule were the most sensitive tools, with a sensitivity of 50% in predicting X4-tropic and 57% in predicting X4-exclusive. However, in comparison to G2P_FPR15, performance of Garrido's rule was better in terms of specificity (93% vs 84%). With regard to HIV-1 subtype, for 7/9 predictive tools specificity for predicting R5-tropic virus was comparable for both CRF02_AG (87% to 100%) and non-CRF02_AG (83% to 100%) subtypes. However, decreased specificity from more than 85% in CRF02_AG subtypes to 75% for non-CRF02_AG subtypes was observed with G2P_FPR10 and G2P_FPR15. In contrast, prediction of X4-exclusive viruses, was about two times less sensitive for CRF02_AG compared with non-CRF02_AG viruses (Table 1).

**Table 1:** Performance of Genotypic approaches to predict tropism of HIV-1 strains circulating in Cameroon.

	All virus populations (n = 55)			CRF02_AG subtype (n = 41)			Non CRF02_AG subtypes (n = 14)	
Predictive tools	Specificity (%)	Sensitivity (%, X4-tropic)	Sensitivity (%, X4-exclusive)	Specificity (%)	Sensitivity (%, X4-tropic)	Sensitivity (%, X4-exclusive)	Specificity (%)	Sensitivity (%, X4-exclusive)
G2P (FPR5)	95.3	33.3	42.9	100.0	20.0	20.0	83.3	100.0
G2P (FPR10)	86.0	41.7	57.1	90.3	30.0	40.0	75.0	100.0
G2P (FPR15)	83.7	50.0	57.1	87.1	40.0	40.0	75.0	100.0
PSSMSINSI	93.0	41.7	42.9	93.5	30.0	20.0	91.7	100.0
PSSMX4R5	86.05	33.3	42.9	87.1	20.0	20.0	83.3	100.0
Position 11/25	93.0	33.3	28.6	90.3	30.0	20.0	100.0	50.0
Net charge	97.7	25.0	42.9	96.8	10.0	20.0	100.0	100.0
Delobel's rule	97.7	33.3	42.9	93.7	11.1	20.0	100.0	100.0
Garrido's rule	93.0	50.0	57.1	93.0	40.0	40.0	100.0	100.0

FPR; false positive rate. X4-exclusive include only viruses using exclusively CXCR4 as a co-receptor.

## Discussion

In this study, sequence of the partial env gene encompassing the V3 region of fifty five samples from Cameroon was analyzed. The genetic strain assigned to 74.5% of them was the recombinant form CRF02_AG. This rate was higher than the 60 to 66% previously reported in Cameroon [13], [14], [17], [33]–[36]. However, it was comparable to the partial env based prevalence of 72.3% reported in blood donor in Cameroon [33]. 9.1% of the sequences analyzed were assigned to CFR22_01A1. This was higher than the 5.5% or 6.6% respectively reported in rural population and blood donors [13], [14]. In our sample set, subtypes A, G, F2 and the recombinant form CRF11_cpx were also present with a rate comparable to those reported in previous studies (ranging from 2 to 4%). Several reports of HIV-1 epidemiology have shown differences in prevalence of strains co-circulating in Cameroon. These differences were mainly related to the sample population and/or to the viral region analyzed. Nevertheless, most of them have shown a high genetic diversity of HIV-1 in this country. Although our study was not designed to describe prevalence of HIV-1 strains in Cameroon, the diversity highlighted here confirms the complexity of HIV-1 epidemic and its dynamic throughout the population. It is well known that HIV-1 genetic diversity has a profound impact on many aspects of the pandemic including diagnostics, vaccine development and response to antiretroviral therapy. The future prospect of including CCR5 antagonists as a treatment option for management of HIV-1 infection in Sub-Saharan countries raises concern about the reliability of laboratory methods used to determine tropism of non-B subtypes and CRFs.

In this study, we have shown that genotypic methods were highly specific in predicting R5-tropic variants. There was no significant difference in specificity of most genotypic methods for CRF02_AG and non-CRF02_AG strains. Our results were consistent with previous studies reporting specificity of over 80% in non-B subtypes and CRFs [32], [37], [38]. Along with specificity, sensitivity of a test to detect X4-tropic viruses is important in clinical settings. Indeed, it has been shown that patients undergoing therapy with CCR5 antagonists may experience virological failure due to the pre-existence of X4-tropic variants [39]–[41]. In our study, sensitivity of CRF02_AG virus populations was extremely low compared to other circulating viruses. Using WebPSSM, the observed sensitivity was no greater than 30% among CRF02_AG strains. This rate was lower than the 70% reported by Raymond et al. [37], but comparable to those reported by Esbjornsson et al. [42]. Although sensitivity rates found in our study were slightly lower than those previously reported, our results were in agreement with the fact that Geno2pheno failed to detect most non-B subtype X4-tropic viruses [6], [43]. The sensitivity of predictive tools was not significantly improved (25% to 33%) by using new simple rules such as 11/25 or net charge. However, it was enhanced to 50% when they were combined with the Garrido rule. This result was lower than the 58% reported by Seclen et al. [32]. However, sensitivity rates in non-B subtypes of genotypic approaches seem to vary between studies of HIV-1 non-B subtypes [31], [32], [37], [43], [44]. This lack of consistency highlights the need for further studies including testing a larger number of samples using both genotypic and phenotypic methods for paired comparative to assess performance of genotypic assays. In our dataset, we observed that R5X4 virus populations were mostly misclassified as R5-tropic variants by genotypic approaches. This finding could be attributed to the high variability of the V3 loop and to the presence of minority X4-tropic variants. In summary, genotypic methods performed relatively well overall in predicting R5-tropic viruses of clinical isolates regardless of HIV-1 subtype. But, sensitivity of these methods for prediction of diverse HIV-1 strains needs to be improved. In our study, with a specificity of 93% and a sensitivity of 50%, the Garrido's rule provide the most optimal balance between specificity and sensitivity for predicting R5 or X4 tropism of HIV-1 strains circulating in Cameroon. It has been demonstrated that HIV infection involving the CXCR4 co-receptor requires CXCR4-associated sequences in the V3 region in combination with other determinants in the gp120 region [45]–[48]. In this context, sequences outside the V3 region which have also been shown to be involved in viral tropism may be worthy of consideration for inclusion into these algorithms in order to improve accuracy of tropism predictive tools.

It should be noted that our findings were obtained using a limited sample size of representative diverse strains and testing a larger number of specimens in this region may be needed to provide more accurate and reliable information about the use of prediction tools for HIV tropism determination of highly diverse HIV-1 strains.

## Conclusions

In light of the emerging and ongoing genetic diversity of HIV in the global AIDS pandemic, optimization of new antiretroviral regimens involving CCR5 antagonists is important. Therefore, evaluation of genotypic prediction of HIV-1 tropism should also include non-B subtypes and CRFs as they are co-circulate in some settings. Our study findings suggest that currently available genotypic methods based on V3 sequence need further optimization for their use with highly diverse strains to accurately predict tropism.
